# A natural frameshift mutation in *Campanula EIL2* correlates with ethylene insensitivity in flowers

**DOI:** 10.1186/s12870-016-0786-4

**Published:** 2016-05-23

**Authors:** Line Jensen, Josefine Nymark Hegelund, Andreas Olsen, Henrik Lütken, Renate Müller

**Affiliations:** Department of Plant and Environmental Sciences, Faculty of Science, University of Copenhagen, Højbakkegård Allé 9-13, 2630 Taastrup, Denmark

**Keywords:** *Campanula*, Deletion, Ethylene, Ethylene insensitive, EIL, EIN, Flower development

## Abstract

**Background:**

The phytohormone ethylene plays a central role in development and senescence of climacteric flowers. In ornamental plant production, ethylene sensitive plants are usually protected against negative effects of ethylene by application of chemical inhibitors. In *Campanula,* flowers are sensitive to even minute concentrations of ethylene.

**Results:**

Monitoring flower longevity in three *Campanula* species revealed *C. portenschlagiana* (*Cp*) as ethylene sensitive, *C. formanekiana* (*Cf*) with intermediate sensitivity and *C. medium* (*Cm*) as ethylene insensitive. We identified key elements in ethylene signal transduction, specifically in *Ethylene Response Sensor 2* (*ERS2*), *Constitutive Triple Response 1* (*CTR1*) and *Ethylene Insensitive 3- Like 1 and 2* (*EIL1* and *EIL2*) homologous. Transcripts of *ERS2, CTR1* and *EIL1* were constitutively expressed in all species both throughout flower development and in response to ethylene. In contrast, *EIL2* was found only in *Cf* and *Cm*. We identified a natural mutation in *Cmeil2* causing a frameshift which resulted in difference in expression levels of *EIL2*, with more than 100-fold change between *Cf* and *Cm* in young flowers.

**Conclusions:**

This study shows that the naturally occurring 7 bp frameshift discovered in *Cmeil2*, a key gene in the ethylene signaling pathway, correlates with ethylene insensitivity in flowers. We suggest that transfer of the *eil2* mutation to other plant species will provide a novel tool to engineer ethylene insensitive flowers.

**Electronic supplementary material:**

The online version of this article (doi:10.1186/s12870-016-0786-4) contains supplementary material, which is available to authorized users.

## Background

Ethylene is a gaseous phytohormone involved in regulating processes of horticultural importance encompassing flower development, fruit ripening, abscission and leaf and flower senescence [1]. In the ethylene signal transduction pathway, ethylene perception is facilitated via a copper co-factor present in receptor proteins integrated in the endoplasmic reticulum (ER) [2, 3]. In *Arabidopsis*, receptor proteins comprising EThylene Response 1 (ETR1) and Ethylene Response Sensor 1 (ERS1) or ETR2, ERS2 and Ethylene INsensitive 4 (EIN4) have been characterised. They differ by the functionality of their kinase domains [4–6]. Ethylene receptors exist as dimers and physically interact with the negative regulator Constitutive Triple Response 1 (CTR1) [7]. The kinase activity of CTR1 is directed towards the C-terminal of Ethylene INsensitive 2 (EIN2), a positive regulator of the ethylene response [8–10]. Ethylene binding to receptors deactivates CTR1 and results in a dephosphorylation of EIN2 [10]. Subsequently, the C-terminal of EIN2 is cleaved and translocated from ER to the nucleus [11, 12] where Ethylene INsensitive 3/Ethylene Insensitive 3-Like (EIN3/EIL)-dependent transcription and activation of the ethylene response occur [13–15].

Postharvest quality of many ornamental plants is sensitive to ethylene during production and distribution [16, 17]. In climacteric plants, flower development is controlled by intrinsic rise in ethylene production and respiration which promotes flower development and senescence. Plant species with climacteric flower senescence are sensitive to exogenous ethylene and may exhibit accelerated petal or flower wilting upon exposure. Commercially important climacteric ornamental plants include carnations, orchids, *Kalanchöe, Campanula* and roses [18–21]. Endogenous ethylene production may arise due to natural floral development but also in response to stress, elevated CO_2_ production [22] or increased auxin production [23]. Hence, ornamental plants are often treated with chemical inhibitors blocking ethylene signaling to improve postharvest quality and prolong flower longevity [24].

Genetic approaches designed to reduce ethylene sensitivity in flowers have modified signaling via the ethylene signal transduction pathway. The *etr1-1* ethylene receptor mutant from *Arabidopsis* fails to bind ethylene [25]. Expression of *etr1-1* in *Petunia* and *Campanula carpatica* flowers [26–28] results in ethylene insensitivity, delayed senescence and postponed flower abscission. Also, transgenic *Petunia* expressing reduced levels of *PhEIN2* displayed delayed flower senescence [29]. However, to date genetic approaches successfully prolonging flower longevity have resulted in transgenic plants [30].

*Campanula* is an economically important ornamental plant, used as indoor potted plant, garden plant, as well as cut flower. The *Campanula* genus consists of approximately 415 species [31]. Here, we characterise expression patterns of flower expressed *ERS*, *CTR* and *EIL* genes in response to floral development and exogenous ethylene in three ornamental species of *Campanula*; *C. portenschlagiana* (*Cp*), *C. formanekiana* (*Cf*) and *C. medium* (*Cm*). The ethylene insensitivity identified in flowers of *C. medium* correlates with the occurrence of a natural mutation in the open reading frame of *EIL2*. This finding holds promise for new breeding strategies towards ethylene insensitive ornamental plants.

## Results

### *Campanula* sensitivity to ethylene

**Fig. 1:**
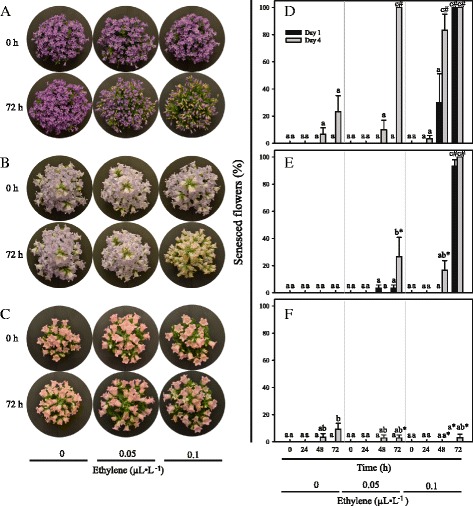
Floral development in response to ethylene exposure in *Campanula*. Visual presentation of flower responses to ethylene concentrations of 0, 0.05 or 0.1 μL · L^−1^ ethylene for 72 h **a** *C. portenschlagiana* (*Cp*), **b** *C. formanekiana* (*Cf*), **c** *C. medium* (*Cm*). Graphical presentation of flower senescence (%) in responses to ethylene concentrations of 0, 0.05 or 0.1 μL · L^−1^ ethylene for 0–72 h **d** *Cp*, **e** *Cf*, **f** *Cm*. Flower senescence were monitored every 24 h for 1-day (black) and 4-day (grey) old flowers. Data are means ± SE. Values with same letters within species and are not significantly different (*P* > 0.05). Values with same symbols between species are not significantly different (*P* > 0.05)

To understand the physiological variation in ethylene sensitivity among *Campanula* species, we used ethylene exposure tests in a postharvest environment. *C. portenschlagiana*, *C. formanekiana* and *C. medium* were selected due to their relevance as ornamental plants. *Cp* was found to be sensitive to ethylene from concentrations of 0.05 μL · L^−1^. Individual flower sensitivity increased with flower age. In *Cp*, old flowers did not survive 0.05 μL · L^−1^ ethylene treatment for 48 h whereas young flowers maintained longevity in a 0.1 μL · L^−1^ ethylene environment for more than 48 h. *Cp* flowers, regardless of age, did not survive after 72 h of the high ethylene treatment (Fig. 1a, d). Less pronounced ethylene sensitivity was found in *Cf* where 26 % of old flowers wilted in response to 72 h of 0.05 μL · L^−1^ ethylene as opposed to 100 % of old *Cp* flowers. Increased ethylene concentration for the same period resulted in complete senescence of 4-day old *Cf* flowers (Fig. 1b, e). As in *Cp*, young *Cf* flowers were less ethylene sensitive than old flowers, however, 93 % of young flowers wilted in response to 0.1 μL · L^−1^ ethylene (Fig. 1e). Thus, flowers of neither *Cp* nor *Cf* could tolerate 72 h of 0.5 μL · L^−1^ ethylene, regardless of flower age. In contrast, *Cm* flowers were non-responsive to ethylene, they maintained both colour and turgor for 72 h in the 0.1 μL · L^−1^ ethylene environment (Fig. 1c, f).

### Identification of key genes in ethylene signal transduction

Degenerate primers were used to identify expressed components in the ethylene signal transduction pathway in *Campanula* flowers. This allowed identification of partial homologs for *ERS2*, *CTR1* and *EIL. Campanula* transcript fragments of *ERS2* and *CTR1* were translated to protein and named according to their closest relative in *Arabidopsis* (Additional files 1 and 2) whereas *Campanula EIN3*/*EIL* homologs were named *EIL1* and *EIL2*. Protein alignment of *Campanula* ERS2 showed high similarity within species in the identified region whereas *Campanula* CTR1 proteins differed (Additional files 1 and 2). In *Cf* and *Cm*, *ERS2* was encoded by a single gene whereas *Cp ERS2* was represented by two loci containing different introns but resulting in identical partial transcripts (Additional file 3). Sequencing showed some polymorphisms among the partial *CTR1* transcripts. These could not be separated in RT-PCR reactions and may represent different alleles in the same locus.

**Fig. 2:**
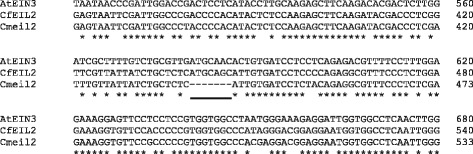
Alignment of *EIL2* gDNA from *Campanula*. The 65 bp fragments of *EIL2* gDNA span the region where *C. medium* (*Cm*) contains a 7 bp deletion and C. *formanekiana* (*Cf*) does not. As a reference the closest ortholog *AtEIN3* from *Arabidopsis thaliana* is included [Genbank: O24606.1]*.* The position of the deletion in *Cmeil2* is underlined. The alignment was produced in Clustal Ω [62]

Also *EIL* transcripts were identified by degenerate primers using flower cDNA as template. Sequencing identified two partial *EIL1* homologs *EIL1a* and *EIL1b* in *Cp* and only one partial *EIL1* homolog in *Cf* and *Cm*. In *Cf* the cDNA pool contained an additional *EIL* homolog, *EIL2*, however this transcript was not readily detectable in *Cm* cDNA using the degenerate primers. As *Cf* and *Cm* have different sensitivities towards ethylene (Fig. 1e, f), primers were designed to separate and amplify both *EIL1* and *EIL2* fragments from *Cf* and *Cm* genomic DNA. Interestingly, *Cmeil2* was found to contain a deletion of 7 bp in the *EIL2* ORF resulting in a frame shift in the corresponding protein (Fig. 2). The 7 bp deletion in *Cmeil2* was verified from independent gDNA extractions (data not shown). At the nucleotide level *CfEIL2* and *Cmeil2* shared 96 % identity to each other and 76 % identity to *CfEIL1* and *CmEIL1* respectively (Table 1). PCR reactions specific for *EIL2* using *Cp* gDNA or cDNA did not amplify a product.

**Table 1:** Percent identity matrix of partial *EIL* nucleotide sequences from *Campanula*

	*CpEIL1a*	*CpEIL1b*	*CfEIL1*	*CmEIL1*	*CfEIL2*	*Cmeil2*
*CpEIL1a*	100					
*CpEIL1b*	93.06	100				
*CfEIL1*	88.49	86.95	100			
*CmEIL1*	88.83	87.29	96.28	100		
*CfEIL2*	74.24	75.13	75.80	76.48	100	
*Cmeil2*	73.08	73.80	74.14	75.51	96.31	100

The identity matrix was produced by Clustal Ω [62]

*Abbreviations*: *Cp C. portenschlagiana*, *Cf C. formanekiana*, *Cm C. medium*

### Expression analysis of putative *ERS2*, *CTR1*, *EIL1* and *EIL2* homologues

**Fig. 3:**
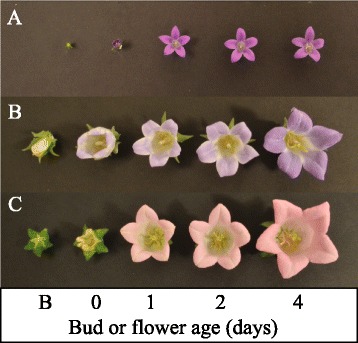
Developmental stages of *Campanula* flowers*.* Developmental stages are unripe bud (B), day 0 (one day before flowering) and 1, 2 and 4-day old flowers of **a** *Campanula portenschlagiana* (*Cp*), **b** *C. formanekiana* and **c** *C. medium* (*Cm*)

**Fig. 4:**
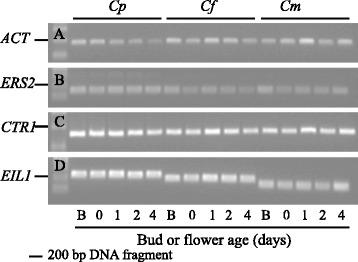
Expression of *ERS2* (**b**), *CTR1* (**c**) and *EIL1* (**d**) in *Campanula* during flower development. As reference gene *Actin* (*ACT* (**a**)) from *Campanula* was used. The developmental stages from bud to flower were; bud (B), flower the day before opening (0), 1-day old flower (1), 2-days old flower (2) and 4-days old flower (4). The presented *Campanula* species are *C. portenschlagiana* (*Cp*), *C. formanekiana* (*Cf*) and *C. medium* (*Cm*)

**Fig. 5:**
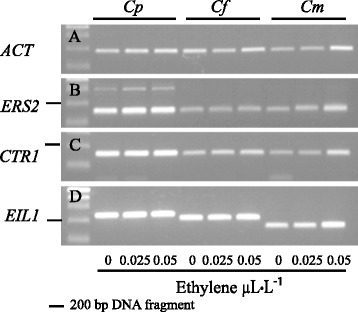
Expression of *ERS2* (**b**), *CTR1* (**c**) and *EIL1* (**d**) in *Campanula* in response to transient ethylene exposure. As reference gene *Actin* (*ACT* (**a**)) from *Campanula* was used. Ethylene was supplied to sealed glass tanks in the following concentrations; 0 μL · L^−1^ (0), 0.025 μL · L^−1^ (0.025) and 0.050 μL · L^−1^ (0.050) ethylene. The presented *Campanula* species are *C. portenschlagiana* (*Cp*), *C. formanekiana* (*Cf*) and *C. medium* (*Cm*). Flowers were marked the day before flower opening; the experiments started the following day (with 1-day old flowers) and were allowed to proceed for 24 h

To address the transcriptional regulation of the ethylene signal transduction pathway in *Campanula*, flower tissues were harvested at five developmental stages (from bud to fully expanded flower on day 4, Fig. 3). Transcripts of putative *ERS2, CTR1* and *EIL1* were expressed constitutively during flower development (Fig. 4). To determine whether *Campanula ERS2, CTR1* or *EIL1* were responsive to ethylene, transcriptional analysis were performed in young (1-day old) flowers exposed to 0.025 μL · L^−1^ or 0.050 μL · L^−1^ ethylene for 24 h. However, neither *ERS2*, *CTR1* nor *EIL1* transcripts were responsive to the applied ethylene treatments (Fig. 5).

**Fig. 6:**
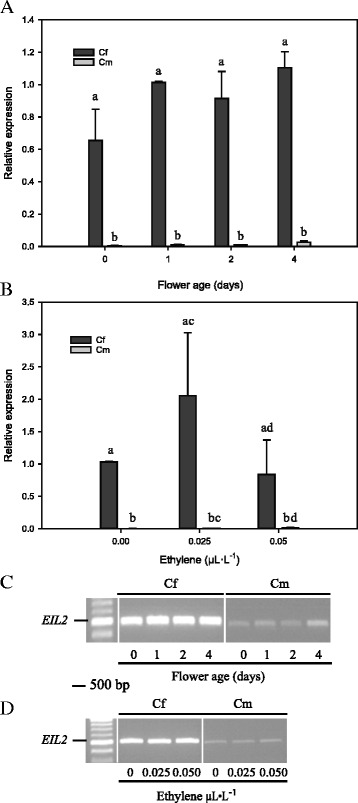
Expression of *EIL2* in large flowered *Campanula* during flower development and following ethylene exposure. *Campanula* species are *C. formanekiana* (*Cf*) and *C. medium* (*Cm*). In **a**, **c** the floral developmental stages were; day before opening (0), 1-day old flower (1), 2-days old flower (2) and 4-days old flower (4). In **b**, **d** ethylene was supplied to sealed glass tanks at the following concentrations; 0.00 μL · L^−1^ (0), 0.025 μL · L^−1^ (0.025) and 0.050 μL · L^−1^ (0.050) ethylene. Results from RT-qPCR are presented as relative expressions in (**a**) and (**b**). The corresponding results of *EIL2* RT-PCR loaded on agarose gels are presented in (**c**) and (**d**). The values of 1-day flowers and 0 μL · L^−1^ ethylene were set to the value 1 in (**a**) and (**b**), respectively. As reference gene *Actin* (*ACT*) from *Campanula* was used, these RT-PCR results are presented in Figs. 4a and 5a. RT-qPCR data were normalized to transcripts of the same *ACT*. Data are means ± SE. Values with same letters are not significantly different (*P* > 0.05)

Expression patterns of *CfEIL2* and *Cmeil2* transcripts were analyzed by classic RT-PCR and RT-qPCR. *CfEIL2* transcripts showed expression pattern and levels similar to those of *CfEIL1* throughout flower development and in response to 0.025 μL · L^−1^ and 0.050 μL · L^−1^ ethylene for 24 h (Fig. 6). In contrast, *Cmeil2* was detectable in trace amounts when analysed by RT-PCR. Quantitative analysis by RT-qPCR showed consistently very low levels of *Cmeil2* through flower development and no transcriptional response to ethylene. Expression levels of *CfEIL2* and *Cmeil2* differed by more than 100-fold in young flowers (day 0, day 1) whereas the same comparison in old flowers (day 4) yielded only 40-fold changes. The variation in fold change was primarily due to non-significant increases in *Cmeil2* transcript levels. Expression levels of *CfEIL2* and *Cmeil2* in response to ethylene were not found to be significantly different due to the large variation in *CfEIL2* expression levels (Fig. 6b).

### The *eil2* frameshift mutation is unique for *C. medium*

**Fig. 7:**
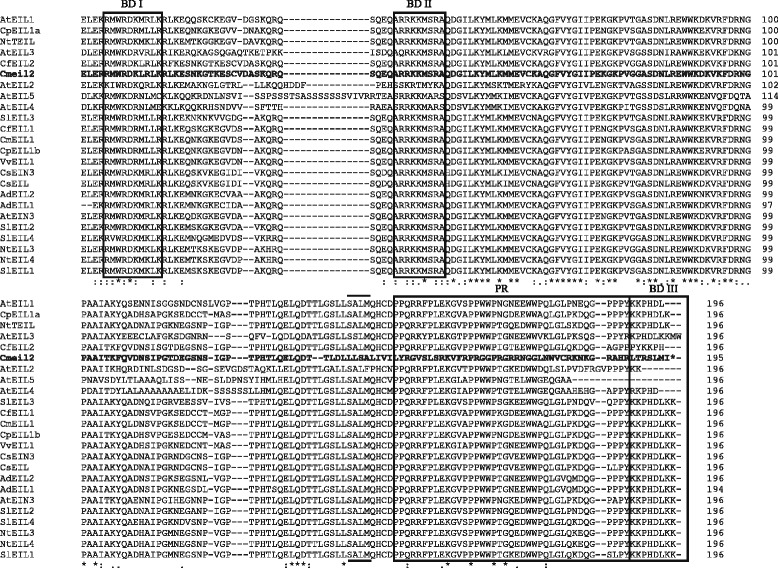
Alignment of partial sequences from translated *Campanula* EIL proteins spanning 196 amino acids. Conserved domains previously described in EIN/EIL proteins are boxed, these are the basic domains (BDI-BDIII) and the proline-rich domain (PR). The conserved SALM motif in which Cmeil2 is mutated is marked with (· · · ·). Conserved aa among all EIN/EILs are marked below with an asterisk. Cmeil2 is presented in bold. The alignment were produced from partial EIN/EIL protein sequences of *C. portenschlagiana* (*Cp*), *C. formanekiana* (*Cf*), *C. medium* (*Cm*), *Actinidia deliciosa* (*Ad*), *Arabidopsis thaliana* (*At*), *Cucumis sativus* (*Cs*), *Nicotiana tabacum* (*Nt*), *Solanum lycopersicum* (*Sl*) and *Vitis vinifera* (*Vv*). Previously named proteins are presented by their species abbreviation followed by their name. The alignment was produced in Clustal Ω [62]

**Fig. 8:**
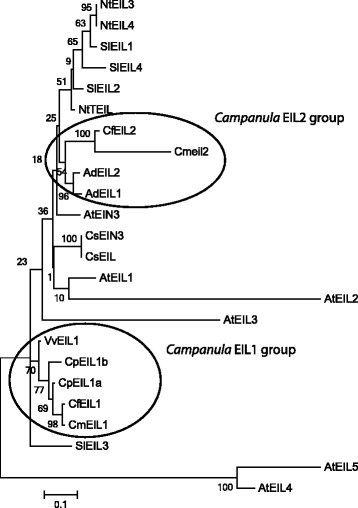
Phylogenetic tree of EILs in plants. The phylogenetic tree was produced from partial EIN/EIL protein sequences spanning 196 amino acids. The clustering in two groups of EIL1 and EIL2 proteins from *Campanula* are highlighted in ellipses. Low bootstrap values in some parts of the phylogenetic analysis are due to the size of the partial EIN/EIL proteins (196 aa) and the high level of aa identity among them. The phylogenetic tree were produced from partial EIN/EIL protein sequences of *C. portenschlagiana* (*Cp*), *C. formanekiana* (*Cf*), *C. medium* (*Cm*), *Actinidia deliciosa* (*Ad*), *Arabidopsis thaliana* (*At*), *Cucumis sativus* (*Cs*), *Nicotiana tabacum* (*Nt*), *Solanum lycopersicum* (*Sl*) and *Vitis vinifera* (*Vv*). Previously named proteins are presented by their species abbreviation followed by their name. The phylogenetic analysis were produced from MEGA version 6 [63]

Alignment of the putative EIL2 protein fragment from *Campanula* with EIL protein sequences from other plants confirmed the presence of three conserved domains found in other EILs. These domains comprise the two basic amino acids binding domains (BD I and BD II) and the proline-rich region (PR) (Fig. 7). At the protein level the putative EILs from *Campanula* were closely related to each other when compared to other EILs except for Cmeil2. Cmeil2 showed high homology to other EILs until the position of the frameshift. The sequence following downstream of the frameshift was only observed in *Cm* and did not show any homology to previously reported EIL proteins. Omission of the deletion from the *Cmeil2* reading frame resulted in a protein that perfectly aligned with other EIL2 proteins (data not shown). Phylogenetic analysis using other plant EILs indicated a close relation among *Campanula* EILs and a clear phylogenetic separation of *Campanula* EIL1 and EIL2 proteins (Fig. 8). Some branch points in the phylogenetic analysis yielded low bootstrap values due to the size of the aligned fragment (200 amino acids) and the high identity among all the EILs (Fig. 8).

### Characterization of *eil2* in *Campanula* species and cultivars

**Fig. 9:**
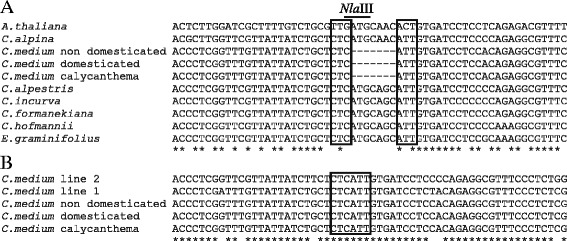
Alignment of genomic *EIL2* DNA sequences. The aligned area presented is centered on the seven bp deletion in *C. medium* (represented with -). Nucleotides bordering the mutation are boxed. Conserved nucleotides are marked with asterisk. **a** *EIL2* from six *Campanula* species, three *C. medium* cultivars, *Edraianthus graminifolius*, and *Arabidopsis EIN3*. The restriction site of *Nla*III found in *EIL2 Campanula* sequences not having the mutation is marked with bold line **b** *eil2* from three *C. medium* cultivars and two PKM breeding lines (line 1 (Sweet Mee®) and line 2)

To elucidate the natural occurrence of *eil2* in *Campanula*, close relatives to *C. medium* were identified as *C. hofmannii*, *C. alpina*, *C. alpestris* and *Edraianthus graminifolius* and *C. incurva* as close relative to *C. formanekiana* [32]. As the *Cmeil2* mutation disrupts an *Nla*III restriction site in *EIL2* a simple screen for the presence of the mutation was developed (Fig. 9). *EIL2* PCR products digested with *Nla*III resulted in either two or three DNA fragments depending on the presence or lack of the *eil2* mutation, respectively. Results obtained via *Nla*III digests were verified by sequencing. Interestingly, *eil2* was found to be specific for *Cm* and did not occur in related *Campanula* species (Fig. 9a). Intraspecific *Nla*III restriction analysis among *Cm* cultivars confirmed the occurrence of *eil2* regardless of cultivar origin (Fig. 9b). Thus the reported frameshift mutation in *Cmeil2* is specific for *Cm* and occurs in all tested *Cm* both among non domesticated and domesticated cultivars.

### Characterization of *eil2* in hybrids of *Cf × Cm*

**Fig. 10:**
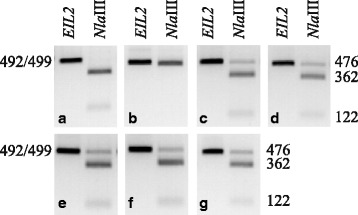
Detection of *EIL2* in hybrids of *C. formanekiana* (*Cf*) and *C. medium* (*Cm*). The original PCR products (*EIL2/eil2*) are 499 bp or 492 bp in *Cf* and *Cm*, respectively. Upon digestion with the restriction enzyme *Nla*III *CfEIL2* produces DNA fragments of 362 bp, 122 bp and 16 bp (**a**). In contrast *Cm* containing the 7 bp deletion in *eil2* produces DNA fragments of 476 bp and 16 bp (**b**). The 16 bp DNA fragments are not detected. *Nla*III restriction analysis of 5 heterozygote *Cf* × *Cm* hybrids shows that all hybrids contain both *Cf* and *Cm* specific DNA fragments (**c**–**g**, hybrids A–E). Hybrids were produced according to [57]

As *Cm* is homozygote for *eil2* whereas *Cf* is homozygote for *EIL2* the performance of *eil2* in heterozygote plants were evaluated by ethylene exposure tests in *C. formanekiana* × *C. medium* hybrids. The presence of *eil2* in *Cf* × *Cm* hybrids (A-E) was verified using the *Nla*III screening system (Fig. 10). Young flowers were exposed to high ethylene concentrations of 5.0 μl · L^−1^ for 72 h and flower responses were scored in categories of no response, signs of senescence and complete senescence (Table 2). Interestingly, four heterozygote *Cf* × *Cm* hybrids showed phenotypes indistinguishable from that of *Cf* with 87–100 % of flowers showing complete senescence in response to ethylene. A single hybrid (E) exhibited an intermediate phenotype with 57 % senesced flowers. In contrast, two *Cm* breeding lines maintained flower longevity longer and only 0–5 % of flowers senesced as a result of 72 h of 5.0 μl · L^−1^ ethylene exposure.

**Table 2:** Flower responses to ethylene exposure in *C. medium*, *C. formanekiana* and *C. formanekiana* × *C. medium* hybrids. Plants were exposed to concentrations of 0 or 5 μl · L^−1^ ethylene for 72 h. Flower responses were grouped in three categories (no senescence, signs of senescence and complete senescence). Data are in % and presented as means ± SE (*n* = 2, except *Cm* line 1; *n* = 4). Values with same letters within treatments in same response are not significantly different (*P* > 0.05)

Species/hybrid	No senescence	Signs of senescence	Complete senescence
	0 μl · L^−1^ ethylene, 72 h		
*C. formanekiana*	87 ± 4.7 ^a^	13 ± 4.7^d^	0.0 ± 0.0^a^
*C. medium* line 1^1^	3.3 ± 2.9^d^	97 ± 2.9 ^a^	0.0 ± 0.0^a^
*C. medium* line 2^1^	53 ± 19 ^ab^	47 ± 19^cd^	0.0 ± 0.0 ^a^
Hybrid A	53 ± 0.0 ^ab^	47 ± 0.0^cd^	0.0 ± 0.0 ^a^
Hybrid B	17 ± 2.4^bcd^	83 ± 2.4 ^ab^	0.0 ± 0.0 ^a^
Hybrid C	40 ± 4.7^bc^	60 ± 4.7^bc^	0.0 ± 0.0 ^a^
Hybrid D	10 ± 7.1^cd^	90 ± 7.1 ^ab^	0.0 ± 0.0 ^a^
Hybrid E	3.3 ± 2.4^cd^	97 ± 2.4 ^ab^	0.0 ± 0.0 ^a^
	5 μl · L^−1^ ethylene, 72 h		
*C. formanekiana*	0.0 ± 0.0 ^a^	0.0 ± 0.0^c^	100 ± 0.0 ^a^
*C. medium* line 1^1^	0.0 ± 0.0 ^a^	95 ± 4.3 ^a^	5.0 ± 4.3^c^
*C. medium* line 2^1^	0.0 ± 0.0 ^a^	100 ± 0.0 ^a^	0.0 ± 0.0^c^
Hybrid A	0.0 ± 0.0 ^a^	13 ± 9.4^c^	87 ± 9.4 ^a^
Hybrid B	0.0 ± 0.0 ^a^	3.3 ± 2.4^c^	97 ± 2.4 ^a^
Hybrid C	0.0 ± 0.0 ^a^	0.0 ± 0.0^c^	100 ± 0.0 ^a^
Hybrid D	0.0 ± 0.0 ^a^	10 ± 7.1^c^	90 ± 7.1 ^a^
Hybrid E	0.0 ± 0.0 ^a^	43 ± 7.1^b^	57 ± 7.1^b^

^1^ *C. medium* breeding lines 1 (Sweet Mee®) and 2

## Discussion

### Ethylene sensitivity in *Campanula*

Ethylene sensitivity of flowers is a recurring problem affecting breeders, producers and costumers of ornamental plants [17]. Thus characterisation of physiological and molecular variations in economically important *Campanula* species are much needed. In the present study, ethylene sensitivity among *C. portenschlagiana*, *C. formanekiana* and *C. medium* were found to depend on physiological and genetic factors. Ethylene sensitivity in flowers were dependent on genotype as *Cp* was highly sensitive, *Cf* had an intermediate level of sensitivity and *Cm* was found to be insensitive to high concentrations of ethylene (Fig. 1, 10). Labelling of flower developmental stage showed a link between flower age and ethylene sensitivity, where a higher proportion of flowers senesced among the older flowers. Only old flowers of *Cp* were sensitive to low amounts of 0.05 μL · L^−1^ ethylene, whereas all flowers of *Cp* were sensitive to 0.1 μL · L^−1^ ethylene. Doubling the ethylene concentration resulted insignificant increase in senesced *Cf* flowers from 26 to 100 % and also in complete loss of flower longevity in young *Cf* flowers (Fig. 1). In the same experimental settings all flowers of *Cm* were insensitive to ethylene. A similar correlation between flower age and ethylene sensitivity has been observed in *Pelargonium peltatum* [33]. This age dependent increase in sensitivity may also be connected to pollination as some plant species induce flower senescence upon pollination [34–36].

Whereas *Cf* flowers are ethylene sensitive in a concentration dependent manner (Fig. 1e, Table 2) not even a 50-fold increase in ethylene concentration reduced flower longevity in *Cm*. This indicates that ethylene insensitivity of *Cm* is independent of ethylene concentrations (Fig. 1f, Table 2).

### Constitutive expression of *ERS2* and *CTR1* in flowers

Expression of *ERS2* was found to be constitutive in *Campanula* during floral development and transcripts in young flowers were also unresponsive to low concentrations of ethylene (Figs. 4b and 5b). In both *Arabidopsis* and roses ethylene receptors are encoded by five genes [6, 37–39], some of which exhibit differential expression in response to exogenous ethylene and are regulated during flower development tissues [40, 41]. Also, exogenously applied ethylene does not affect levels of *Dianthus caryophyllus ERS2* in petals but this gene is regulated by flower development [42]. Collectively, results obtained in other plants indicate that ethylene receptor families comprise multiple members. The genome of *Cp* was found to contain two homologs of *ERS* receptors whereas only one gene/transcript was identified in *Cf* and *Cm*. As gDNA was also used as template in the cloning reactions mRNA levels in flower tissues should not be the determining factor. Thus all three *Campanula* species likely encode additional ethylene receptors which were not identified here due to primer specificities.

Similarly as for *ERS2*, all *Campanula CTR1* transcripts were constitutively expressed during flower development and did not respond to application of exogenous ethylene (Figs. 4c and 5c). The same pattern was observed in roses where *RhCTR1* and *RhCTR2* were constitutively expressed throughout flower development; however both *RhCTR* transcript levels increased in response to ethylene [43]. In some plant species the genomic structure of *CTR1* is highly complex. Banana and tomato *CTR1* exist in a 15 exon 14 intron structure yielding complete ORFs of 11.5 and 12 kb, respectively [44, 45]. Even with a long elongation time in PCR reactions we were not able to identify the full-length sequence of *Campanula CTR1*. However small polymorphisms detected in the partial *Cp*, *Cf* and *Cm CTR1* transcripts indicate that these plants may be heterozygote in the *CTR1* locus or that an additional copy of *CTR1* exists. Two and four *CTR* homologs have been identified in roses and tomato, respectively [43, 46]. Hence an additional *CTR1* in *Campanula* is not unlikely.

### Occurrence and expression of EILs in *Campanula*

The *EIN3*/*EIL* family encodes transcription factors mediating the initialization of the physiological ethylene response [13, 47]. In the three *Campanula* species investigated here approximately 600 bp of a flower expressed *EIL* homologue were identified, *EIL1*. All *EIL1* transcripts were consistently expressed through flower development and did not respond to applied ethylene (Figs. 4d and 5d). In the small flowered *Cp* two close homologs of *EIL1* were identified by sequencing as *EIL1a* and *EIL1b*, however the two could not be separated during expression analysis. In contrast, the two large flowered *Campanula* (*Cf* and *Cm*) both contained the ORF of *EIL2*, a gene not detected in *Cp. CfEIL2* gene expression was like *CfEIL1* constitutive throughout flower development and nonresponsive to exogenous ethylene (Fig. 6c, d). In *Cm*, *eil2* was not expressed due to the deletion of 7 bp in the ORF (Figs. 2 and 6). Translational analysis of the partial Cmeil2 protein indicated that the 7 bp deletion would introduce a frameshift prior to what should have been the proline rich domain in *Cmeil2* (Fig. 7). On nucleotide level *CfEIL2* and *Cmeil2* share 96 % identity (Table 1), showing that they are close homologs. The close homology among *CfEIL2* and *Cmeil2* was supported by phylogenetic analysis (Fig. 8). Translation of the putative *Cmeil2* indicate that the frameshift mutation in *eil2* disrupts the putative major DNA binding domain in EIL2 in front of the proline rich region and simultaneously introduces a stop codon 50 amino acids further downstream in the eil2 protein sequence. Hence, if translated the protein would be truncated to approximately 40 % of the expected size when compared to AtEIN3 (Fig. 7). The position and functionality of EIL DNA binding domains have been characterised in *Arabidopsis* EIL3 and in cucumber EIN3 [48, 49].

Whether the promoters of *CfEIL2* and *Cmeil2* share the same specificity remains to be shown, however *Cmeil2* may have been expressed in flowers at one point as traces of the transcript was observed in RT-PCR and in RT-qPCR (Fig. 6). Finally, additional *EIL* homologs may be present in *Campanula* as 4–6 homologs have been identified in *Arabidopsis*, tomato and tobacco [13, 50–52].

Previous studies of *EIN3*/*EIL* homologs have shown constitutive expression in *Paeonia*, however in *Dianthus caryopyllus DcEIL1/2* and *DcEIL3* transcripts in petals and styles increased rapidly after ethylene treatment of flowers and then gradually declined [53,54]. The constitutive expressions of *ERS2*, *CTR1* and *EILs* in the ethylene signal transduction pathway in *Campanula* indicate that flowers are capable of a fast physiological response in the presence of ethylene. This correlate well with earlier results where *Campanula* has been described as an ethylene sensitive species [20]. Furthermore, the lack of transcriptional response to ethylene exposure could be a combination of regulatory steps on the protein level [55, 56].

### The *Cmeil2* phenotype was inherited as a recessive trait

In the present study, the *eil2* frameshift mutation was identified not only in Danish domesticated *Cm* but also in non domesticated specimens of *Cm* and in the closely related *C. medium* var. *calycanthema* (Fig. 9). This suggests that the *eil2* frameshift must have occurred in an earlier ancestor of *Cm*. None of the closely related *Campanula* species *C. alpina*, *C. alpestris*, *C. hofmannii* or *E. graminifolius* contained *eil2* (Fig. 9). In hybrids of *Cf* × *Cm*, heterozygote *eil2* resulted in very similar ethylene responses as observed in *Cf* as only one of five hybrids showed an intermediate phenotype shifted towards increased ethylene insensitivity when compared to *Cf* (Table 2). This indicated that the *eil2* phenotype was restored by the presence of a wild type *EIL2* and was inherited as a recessive trait. Homozygote *eil2* in *Cf* could not be obtained via crossings as both male and female parts of *Cf* × *Cm* hybrids were sterile. Results obtained here are the first to describe the effects of an *ein3*/*eil* mutation in flowers. Also, no reports exist of approaches where *EIN3*/*EIL* genes have been knocked out or silenced via gene modification. Therefore the *eil2* phenotype described in *Cm* cannot be directly compared to related phenotypes in flowers of other plant species. In *Arabidopsis*, the closest homolog to *Campanula EIL2* is *EIN3* (*AtEIN3*). In *Arabidopsis*, *ein3* mutants are well characterised and they too are inherited in a recessive manner [13]. Also phenotypes in *ein3* or *eil* mutants in *Arabidopsis* are only described in seedlings or mature rosettes. To our knowledge ethylene sensitivity and floral development has not been described in *Arabidopsis ein3* or *eil* mutants. However, in support of our results are data from tomato where expression of *LeEIL1*, *LeEIL2* or *LeEIL3* antisense transcripts results in ethylene insensitive buds [51]. Collectively, this study is the first to correlate ethylene insensitivity in flowers to an *ein*/*eil* mutant phenotype.

### Future approaches to achieve ethylene insensitive plants

Previous studies have indicated that there may be specific functions for the individual *EIL*s. Thus to alter the physiological response of flowers to ethylene, the right ortholog in each plant species has to be identified. In the framework of this study, the *Cmeil2* frameshift mutation could potentially be transferred to *EIL2* homologs of related species by conventional crossing or the deletion could be introduced by wide hybridisation among related species, assisted by embryo rescue techniques when necessary [57].

The frameshift in *Cmeil2* is positioned in a highly conserved region, and it therefore holds potential for molecular breeding towards ethylene insensitive plants. However, the identification of the full genomic sequence in *Cm* and *Cf* and the full sequence of the translated gene product in *Cf* are essential steps in this process. Ultimately, we propose that the identified 7 bp deletion in *Cmeil2* may be used to confer ethylene insensitivity to other plant species. This may be feasible via targeted mutagenesis techniques utilizing ZNF, TALENs [58] or CRISPR/Cas9 [59]. As a result, ethylene insensitivity may be transferred from *Cm* to other important climacteric ornamentals e.g., roses, *Petunia*, carnations, or even to edible climacteric crops such as broccoli and tomato.

## Conclusions

We characterised the physiological and molecular responses among three *Campanula* species to exogenous ethylene. Key genes in the ethylene signal transduction pathway *ERS*, *CTR* and *EIL1* were found to be constitutively expressed in *Campanula* and unresponsive to exogenous ethylene. However, *EIL2* was found to be specific for the large flowered species *C. formanekiana* and *C. medium*, but was not expressed in *Cm* due to a 7 nucleotide frameshift in the coding region of *Cmeil2*. The natural mutation identified here in *Cmeil2* correlates with the observed ethylene insensitivity in this species. This finding holds great potential for future breeding strategies towards ethylene insensitive plants.

## Methods

### Plant materials and growth conditions

*Campanula portenschlagiana* Schultes ‘Blue GET MEE®’ (*Cp*), *Campanula formanekiana* Degen & Doefler ‘Blue MARY MEE®’ (*Cf*), *Campanula medium* L. ‘Sweet MEE®’ (*Cm*), *C. medium* breeding line 2 and *C. medium* var. *calycanthema* were received from the nursery Gartneriet PKM A/S (Odense, Denmark) in a developmental stage with young flower buds. Hybrids of *C. formanekiana* × *C. medium* (A-E) were produced by ovule culture [57] or at Gartneriet PKM A/S. Upon arrival, plants were transferred to a greenhouse with 18 °C day/15 °C night and a 16-h photoperiod of natural light. Seeds of the non domesticated *C. medium* were provided by the Alpine Staudengärtnerei (Leisning, Germany). Seeds of *C. alpina*, *C. alpestris*, *C. hofmannii*, *C. incurva* and *Edraianthus graminifolius* were obtained from B & T World Seeds (Aigues-Vives, France).

### Identification of putative *ERS2*, *CTR1* and *EIL* genes

Based on the NCBI GenBank [60] sequence from *Campanula carpatica* (GenBank: AF413669) intron spanning primers were designed to amplify partial fragments of *ERS2. ERS2* PCR on gDNA produced two *ERS2* products in *Cp* and one *ERS2* genomic fragment in *Cf* and *Cm*. In *Cp*, The two *CpERS2* products were derived from two genes containing different intron sizes but coding for very similar transcripts (*CpERS2a* and *CpERS2b*, Additional file 3). A partial *CTR1* was produced using degenerate primers aligning to conserved areas among *Musa acuminata, Solanum lycopersicon, Arabidopsis thaliana* and *Rosa hybrida CTR1* sequences (GenBank:JF430422, GenBank:AF096250, GenBank:NM_180429, and GenBank:AY032953). Sequencing showed some polymorphisms in *CTR1* of *Cp*, *Cf* and *Cm*. This could indicate that more than one copy of *CTR1* exist in *Campanula*. Partial *EIL* genes cloned via degenerate primers produced from *Malus* x *domestica* and *Solanum lycopersicon EIL* sequences (GenBank:GU732486 and GenBank:NM_001247617). *Campanula ACT* was amplified using degenerate primers, sequenced and from this sequence specific primers were designed for expression analyses. Primers and PCR product sizes are presented in Additional file 3.

Genomic DNA from *Cp, Cf,* and *Cm* was isolated from flowers with DNeasy Plant Mini Kit (Qiagen) using 300 mg of plant material following manufacturer’s recommendations*.* PCR reactions used 100–250 ng gDNA, 2 % (v/v) DMSO and polymerase LaTaq (Takara Bio Inc.) as manufacturer recommends. Reactions followed the program; 4 min 94 °C, 33–35 cycles of [30 s 94 °C, 1 min 60 °C, 1 min 72 °C] and a final 7 min elongation step at 72 °C in a MyCycler (Biorad). Cloning of PCR-products was via TOPO TA Cloning® kit (Life Technologies Corp, Invitrogen) as recommended by manufacturer. Plasmids were purified by QIAprep Spin Miniprep kit (Qiagen) and sequenced by Eurofins MWG Operon. *EIL2* PCR products were purified with QIAquick PCR purification Kit (Qiagen) and restriction analyses using *Nla*III were done as supplier recommends (New England Biolabs).

### Ethylene exposure experiments

To monitor flower development, individual buds were labelled one day before flower opening. This stage was termed day 0. In the following days newly opened flowers (day 1) and 4 days old flowers (day 4) were identified, tagged and used in subsequent experiments. This allowed two morphologically different stages to be monitored simultaneously throughout the ethylene exposure experiments. Ethylene exposure were conducted in a climate chamber in glass tanks with postharvest growth conditions; 20 °C day/18 °C night, 16-h photoperiod at 10–12 μmol m^−2^ · s^−1^ provided by cool-white fluorescent tubes (Philips Master TL-D-36 W/830). Each glass tank had a volume of 128 L and contained three plants. Flower labeling resulted in each glass tank containing three plants with a total of 15 labelled flowers for each developmental stage (day 1 and day 4). Except for *Cm* where 8–13 labeled flowers pr. growth stage were used. Ethylene concentrations of 0 μL · L^−1^, 0.05 μL · L^−1^ or 0.1 μL · L^−1^ were obtained by injection of gaseous ethylene (Mikrolab Aarhus A/S) into sealed glass tanks. Flowers were monitored, tanks ventilated and ethylene reinjected every 24 h. For Fig. 1 a senescent flower was defined as a flower showing twisted or closed corolla or wilted. For Table 2, ethylene sensitivity of *Cf* × *Cm* hybrid flowers were classified in three categories; no symptoms, signs of senescence (partial wilting and discoloration of corolla) and complete senescence (complete wilting and full discoloration of corolla). The latter experiments were done in glass tanks with 5 μl · L^−1^ ethylene for 72 h. *Cp* and *Cf* experiments were repeated twice whereas *Cm* experiments were in three replicates.

Gene expression analyses were done at developmental stages: bud, day 0 (one day before flowering), day 1, day 2 and day 4 (Fig. 3). Plants were subjected to low ethylene concentrations of 0.025 μL · L^−1^ and 0.050 μL · L^−1^ ethylene for 24 h. Each tank contained three plants with labeled 1-day flowers. Flowers from each tank were pooled, harvested in liquid nitrogen, grinded and used for RNA extraction. Each experiment was performed in three replicates.

### RNA extraction, cDNA synthesis and expression analysis

RNA was extracted using RNeasy Plant Mini kit (Qiagen) with the following change to manufacturer’s protocol: Cell lysis were done using RLT buffer with 0.01 % β-mercaptoethanol (v/v) for 1 min at 56 °C. RNA yield and purity (A260/A280 ratio > 2.0) was estimated by a Nanodrop^TM^ 1000 Spectrophotometer (Thermo Fisher Scientific Inc.). RNA integrity was evaluated on 1.2 % agarose gels. Purified RNA was stored at -80 °C. RNA was DNase treated with Amplification Grade DNase I (Invitrogen) and cDNA synthesis was done using iScript cDNA Synthesis kit as recommended (Biorad). In 20 μl reactions 0.8 μg RNA was used. No contamination of DNA in cDNA was verified in non RT samples. Expression analyses were done using 5-fold diluted cDNA and ExTaq as polymerase as recommended (Takara Bio Inc.) in MyCycler (Biorad). Primers and gene specific reaction settings used in expression analysis are presented in Additional file 4. RT-PCR program; 4 min 94 °C, 25–32 cycles of [30 s 94 °C, 1 min 55 °C, 1 min 72 °C] and 7 min 72 °C. Reactions for RT-qPCR were performed on an ICycler instrument (Bio-Rad) by using the iQ SYBR Green Supermix (Bio-Rad) according to supplier’s instructions [61] using the program; 95 °C for 10 min, 50 cycles of [30 s at 95 °C, 1 min at 57.5 °C and 1 min at 72 °C]. To evaluate the efficiency of qPCR, serial dilutions of cDNA were used to generate a standard curve. This resulted in R^2^ values of 0.998 and 0.994 for *ACT* and *EIL2* primer sets, respectively. Threshold cycles (Ct), (defined as cycle were the signal exceeds ten times the standard deviation of the baseline), for *CfEIL2* and *Cmeil2* were standardized to the corresponding *Actin* Ct (ΔCt). The relative quantification of target gene *CfEIL2* and *Cmeil2* between the different treatments was determined as 2^(−ΔΔCt). Values are based on three replicates.

### Bioinformatics and statistics

Sequence identification and analysis were done using CLC sequence viewer (CLC bio), BLAST and Clustal Ω [62]. Phylogenetic analysis were conducted using MEGA version 6.06 [63]. Statistical analyses were done in SigmaPlot v. 13 by one way analysis of variance using the Holm-Sidak method.

## Additional files

Additional file 1: Alignment of partial sequences from translated *Campanula* ERS2 proteins. As a reference *Arabidopsis thaliana* (At) ERS2 is included [Genbank: P93825]. The first amino acids position in *Arabidopsis* cds is presented in brackets. Abbreviations are *C. portenschlagiana* (Cp), *C. formanekiana* (*Cf*) and *C. medium* (Cm). Consensus among *Arabidopsis* and *Campanula* are marked with asterisk. The boxed areas enclose identical aa among *Campanula* ERS2. The alignment was produced in Clustal Ω [62]. (PDF 218 kb) Additional file 2: Alignment of partial sequences from translated *Campanula* CTR1 proteins. As a reference *Arabidopsis thaliana* CTR1 aa 424–663 are included [Genbank: NP850760]. Abbreviations are *C. portenschlagiana* (*Cp*), *C. formanekiana* (*Cf*) and *C. medium* (*Cm*). Consensus among *Arabidopsis* and *Campanula* are marked with an asterisk. The alignment was produced in Clustal Ω [62]. (PDF 209 kb) Additional file 3: Primers and PCR product sizes used for identification of sequences homologue to *ERS2*, *CTR1*, *EIL* and *Actin* in *Campanula*. (PDF 224 kb) Additional file 4: Primers used for expression analysis of *ERS2*, *CTR1*, *EIL1*, *EIL2* and *Actin*. (PDF 191 kb)

## Abbreviations

1-MCP: 1-methylcyclopropene
*Cf*: *Campanula formanekiana*
*Cm*: *Campanula medium*
*Cp*: *Campanula portenschlagiana*
CTR: constitutive triple response
EIL: ethylene insensitive3-like
ERS: ethylene response sensor
RT-qPCR: quantitative reverse transcription PCR
