# Supplementary material for: A natural frameshift mutation in Campanula EIL2 correlates with ethylene insensitivity in flowers
Source: BMC Plant Biol. 2016 May 23;16:117. doi: 10.1186/s12870-016-0786-4 (PMC4877742; doi:10.1186/s12870-016-0786-4)
Supplement: Additional file 1: — Alignment of partial sequences from translated Campanula ERS2 proteins. As a reference Arabidopsis thaliana (At) ERS2 is included [Genbank: P93825]. The first amino acids position in Arabidopsis cds is presented in brackets. Abbreviations are C. portenschlagiana (Cp), C. formanekiana (Cf) and C. medium (Cm). Consensus among Arabidopsis and Campanula are marked with asterisk. The boxed areas enclose identical aa among Campanula ERS2. The alignment was produced in Clustal Ω [62]. (PDF 218 kb) [file 12870_2016_786_MOESM1_ESM.pdf]

```
AtERS2 (330)  ELEIVKVVAD QVAVAISHAV ILEESQLMRE KLAEQNRALQ VARENALRAN QAKAAFEQMM
CpERS2b       ----VEVVAD QVAVALSHAA ILEESMRARD QLMEQNIALD LARQEAEMAI QARNDFLA--
CmERS2        ----VEVVAD QVAVALSHAA ILEESMRARD QLMEQNIALD LARQEAEMAV RARNDFLA--
CpERS2a       ----VEVVAD QVAVALSHAA ILEESMRARD QLMEQNIALD LARQEAELAI RARNDFLA--
CfERS2        ----VEVVAD QVAVALSHAA ILEESMRARD QLMEQNIALD LARQEAEMAV RARNDFLA--
                  *:**** *****:***. *****   *: :* *** **: :**::*  *   *:   *
```

Additional file 1. Alignment of partial sequences from translated *Campanula* ERS2 proteins. As a reference *Arabidopsis thaliana* (At) ERS2 is included [Genbank: P93825]. The first amino acid position in the *Arabidopsis* cds is presented in brackets. Abbreviations are *C. portenschlagiana* (Cp), *C. formanekiana* (Cf) and *C. medium* (Cm). Consensus among *Arabidopsis* and *Campanula* are marked with asterisk. The boxed areas enclose identical aa among *Campanula* ERS2. The alignment was produced in Clustal Ω [62].
