# Supplementary material for: A natural frameshift mutation in Campanula EIL2 correlates with ethylene insensitivity in flowers
Source: BMC Plant Biol. 2016 May 23;16:117. doi: 10.1186/s12870-016-0786-4 (PMC4877742; doi:10.1186/s12870-016-0786-4)
Supplement: Additional file 2: — Alignment of partial sequences from translated Campanula CTR1 proteins. As a reference Arabidopsis thaliana CTR1 aa 424–663 are included [Genbank: NP850760]. Abbreviations are C. portenschlagiana (Cp), C. formanekiana (Cf) and C. medium (Cm). Consensus among Arabidopsis and Campanula are marked with an asterisk. The alignment was produced in Clustal Ω [62]. (PDF 209 kb) [file 12870_2016_786_MOESM2_ESM.pdf]

```
CpCTR1        SILVSSPLCHPRFRAVEAGENFRMLAELYFRDGQSLNIAFDDASSGIVTD---------QEDGTELLQR-S--KMNQNDR  68
CfCTR1        SISISSPLRFPRFRQVEPTIDFRALAKQYFSDCESLNIVFDDPSTGDG------------ASGDAIYPKIG--QPKSHDR  66
CmCTR1        SISISSPLRFPRFRQVEPTIDFRALAKQYFSDCESLNIVFDDPSTGDG------------ASGDAIYPKIG--QPKSHDR  66
AtCTR1        SISISSPLRFPRPKPVEPAVDFRLLAKQYFSDSQSLNLVFDPASDDMGFSMFHRQYDNPGGENDALAENGGGSLPPSANM 502
              ** :**** .** : **.  :** **: ** * :***:.** .* .              ..  :  . .     . :

CpCTR1        NAH-TPFSGNNLE--TSTSALPQKAAWPFNQVRGM-QMNG--LCRRVPNATSSTQIANNATSSNLLSLDQRNAQEISPFS 142
CfCTR1        DPQ-LPRAYNVTN--VMNAPNVVKDVVPLKNTRQVGHRDGPPFIALAD-------PRMGAT-NNDQRFTEGGGQLVSFN- 134
CmCTR1        DPR-LPRAYNVTN--VMNAPNVVKDVVPLKNTQQVGHRDGPPFIALAD-------PRMGAT-NNDRRFTEGGGQLVSSI- 134
AtCTR1        PPQNMMRASNQIEAAPMNAPPI--------------------------------------------------SQPVPNR- 531
               .:    : *  :    .:.                                                    .* :.

CpCTR1        DRRHDSHKLYF-EEDLDIHWDDLDLKEKIGKGSFGTVHRAEWKGMEVAVKVLMEQDFHAERFKEFLMEVAIMKRLRHPNI 221
CfCTR1        ----PSREITFDVEDLDIPWSDLILKERIGSGSFGTVHRADWNGSDVAVKILMEQDLHAERFTEFLREVAIMRRLRHPNI 210
CmCTR1        ----PSREITFDVEDLDIPWSDLILKERIGSGSFGTVHRADWNGSDVAVKILMEQDLHAERFTEFLREVAIMRRLRHPNI 210
AtCTR1        ----ANRELGLDGDDMDIPWCDLNIKEKIGAGSFGTVHRAEWHGSDVAVKILMEQDFHAERVNEFLREVAIMKRLRHPNI 607
                   .::: :  :*:** * ** :**:** *********:*:* :****:*****:****..*** *****:*******

CpCTR1        VLFVGAVTQPPNLSIVTECLSRGSLFKLLHLPNADFILDETLRLNMAYDV----- 271
CfCTR1        VLFMGAVTQPPNLSIVTEYLSRGSLYRLLHKPGPKEVLDERRRLSMAYDVAKRNE 265
CmCTR1        VLFMGAVTQPPNLSIVTEYLSRGSLYRLLHKPGPKEVLDERRRLSMAFDVANGMN 265
AtCTR1        VLFMGAVTQPPNLSIVTEYLSRGSLYRLLHKSGAREQLDERRRLSMAYDVAKGMN 662
                ***:************** ******::*** ...   ***  **.**:**
```

Additional file 2. Alignment of partial sequences from translated *Campanula* CTR1 proteins. As a reference *Arabidopsis thaliana* CTR1 aa 424-663 are included [Genbank: NP850760]. Abbreviations are *C. portenschlagiana* (Cp), *C. formanekiana* (Cf) and *C. medium* (Cm). Consensus among *Arabidopsis* and *Campanula* are marked with an asterisk. The alignment was produced in Clustal Ω [62].
