# Supplementary material for: A natural frameshift mutation in Campanula EIL2 correlates with ethylene insensitivity in flowers
Source: BMC Plant Biol. 2016 May 23;16:117. doi: 10.1186/s12870-016-0786-4 (PMC4877742; doi:10.1186/s12870-016-0786-4)
Supplement: Additional file 3: — Primers and PCR product sizes used for identification of sequences homologue to ERS2, CTR1, EIL and Actin in Campanula. (PDF 224 kb) [file 12870_2016_786_MOESM3_ESM.pdf]

Additional file 3. Primers and PCR product sizes used for identification of sequences homologue to *ERS2*, *CTR1*, *EIL* and *Actin* in *Campanula*.

Gene	Forward	Reverse	Product size	
			gDNA	cDNA
CpERS2a/b	5’-TGTGGAAGTTGTTGCTGACC-3’	5’-AGCTAGGAAATCATTACGAGCA-3’	305bp/461 bp[a]	163 bp/163 bp
CfERS2	5’-TGTGGAAGTTGTTGCTGACC-3’	5’-AGCTAGGAAATCATTACGAGCA-3’	298 bp	163 bp
CmERS2	5’-TGTGGAAGTTGTTGCTGACC-3’	5’-AGCTAGGAAATCATTACGAGCA-3’	298 bp	163 bp
CpCTR1	5’-CGAATTGCCAARGGMTGTAA-3’	5’-ATTCATYCCGTTTGCCACATC-3’	na	974 bp
CfCTR1	5’-CGAATTGCCAARGGMTGTAA-3’	5’-ATTCATYCCGTTTGCCACATC-3’	na	943 bp
CmCTR1	5’-CGAATTGCCAARGGMTGTAA-3’	5’-ATTCATYCCGTTTGCCACATC-3’	na	943 bp
CpEIL1a/b	5’-WGAGCTMGAGAGGAGGATGTG-3’	5’-GCCTTCTTCAGATCATGAGGC-3’	594 bp/591 bp	594 bp/591 bp
CfEIL1	5’-WGAGCTMGAGAGGAGGATGTG-3’	5’-GCCTTCTTCAGATCATGAGGC-3’	591 bp	591 bp
CmEIL1	5’-WGAGCTMGAGAGGAGGATGTG-3’	5’-GCCTTCTTCAGATCATGAGGC-3’	591 bp	591 bp
CfEIL2	5’-WGAGCTMGAGAGGAGGATGTG-3’	5’-GCCTTCTTCAGATCATGAGGC-3’	603 bp	603 bp
Cmeil2	5’-WGAGCTMGAGAGGAGGATGTG-3’	5’-GCCTTCTTCAGATCATGAGGC-3’	596 bp	596 bp
ACTIN	5’-GCAGGACGTGATCTGACTGA-3’	5’-GGGAACATRGTTGAWCCACCAC-3’	380 bp	na

[a]In *Cp* the ERS2a and ERS2b genes contain different intron sizes, however the resulting transcripts spanning these regions do not contain any differences in size.
