# Supplementary material for: A natural frameshift mutation in Campanula EIL2 correlates with ethylene insensitivity in flowers
Source: BMC Plant Biol. 2016 May 23;16:117. doi: 10.1186/s12870-016-0786-4 (PMC4877742; doi:10.1186/s12870-016-0786-4)
Supplement: Additional file 4: — Primers used for expression analysis of ERS2, CTR1, EIL1, EIL2 and Actin. (PDF 191 kb) [file 12870_2016_786_MOESM4_ESM.pdf]

Additional file 4. Primers used for expression analysis of *ERS2*, *CTR1*, *EIL1*, *EIL2* and *Actin*.

**Transcript**	**Forward**	**Reverse**	**Product size**
ERS2	5’-TGTGGAAGTTGTTGCTGACC-3’	5’-AGCTAGGAAATCATTACGAGCA-3’	163 bp
CTR1	5’-GTTCGATTTGGGATTGACAGG-3’	5’-CCAATGACCTGAAATCAATGGTTG-3’	168 bp
CpEIL1a/b	5’-GCGAGGACTGCTGTACGATG-3’	5’-GCCTTCTTCAGATCATGAGGC-3’	243 bp
CfEIL1	5’-ACCTCACACATTGCAGGAA-3’	5’-GCCTTCTTCAGATCATGAGGC-3’	215 bp
CmEIL1	5’-CAGCTTTGATGCAGCATTGTG-3’	5’-GCCTTCTTCAGATCATGAGGC-3’	165 bp
CfEIL2	5’-GGGGACGAAGGAATCATGTGTT-3’	5’-CGCCCCTTGATCCTTCTGCAAA-3’	498 bp
Cmeil2	5’-GGGGACGAAGGAATCATGTGTT-3’	5’- CGCCCCTTGATCCTTCTGCAAA-3’	491 bp
ACT	5’-CACTACAACTGCCGAACGG-3’	5’-CGAAGGCTGGAATAGGACCTC-3’	195 bp
